# Effects of maternal dietary energy restriction on breast muscle fibre development in the offspring of broiler breeders

**DOI:** 10.5713/ab.20.0712

**Published:** 2021-04-23

**Authors:** Hongzhi Wu, Hao Sun, Chengzhan Ma, Lina Lian, Lei Lu, Liangmei Xu, Li Xu

**Affiliations:** 1 College of Animal Science and Technology, Northeast Agricultural University, Harbin, Heilongjiang 150030, China; 2 Hebei Sogreen Food Co., Ltd. Shijiazhuang, Hebei, 050000, China

**Keywords:** Breast Muscle Fiber, Broiler Breeder, Energy Restriction, Myostatin

## Abstract

**Objective:**

The effects of maternal dietary energy levels on breast muscle fibre development in offspring of broiler breeders were investigated.

**Methods:**

A total of 480 20-week-old Arbor Acres (AA) healthy female broiler breeders, with an average body weight of 2.33±0.01 kg, were randomly divided into 4 treatment groups with 6 replicates and 20 broiler breeders for each replicate and fed a corn and soybean meal diet with 100%, 80%, 70%, and 50% energy levels, respectively. Approximately 300 eggs per treatment were collected for incubation for 6 days. Then, 120 0-day-old female broilers at each energy level were randomly selected and divided into 6 replicates with 20 broilers for each replicate, with this experimental phase with the offspring lasting for 49 days.

**Results:**

Compared with the 100% energy group, the breast muscle fibre diameter at embryonic day 21 in the 80% energy group was significantly reduced (p<0.05). In the 80% energy group, the muscle fibre density of the breast increased significantly (p<0.05) at embryonic days 15 and 21. The breast muscle fibre diameter of the offspring in each group was significantly decreased (p<0.05) on the 1st day. The breast muscle sarcomere length of the embryos in the 80% energy group was significantly higher (p<0.05) than those in the 70% and 50% energy groups. Compared with the 100% energy group, the expression of the myostatin gene in the offspring was significantly decreased (p<0.05).

**Conclusion:**

In conclusion, the effects of a maternal dietary energy level of 80% in this study were found to be optimal for breast muscle fibre development in offspring, which indicated that the metabolic energy level of AA broilers of 9.36 MJ/kg for the mid-term diet for laying eggs has a more practical significance.

## INTRODUCTION

Unlike mammals, avian embryos are not directly connected to the mother, and the genetic and nongenetic information of the offspring is deposited in the egg [1]. Maternal nutrition is an essential factor affecting the development of muscle fibres in offspring [2]. The embryo stage is the critical period of development of animal muscle fibres [3]. In poultry, the number of muscle fibres has been determined before hatching. The increase in muscle mass after birth is mainly caused by the fusion of muscle fibres’ satellite cells [4]. The number of muscle fibres has been determined before birth in animals, and during postnatal growth and development, mainly the muscle fibre diameter changes, which is primarily caused by the growth of myofibrils [5]. A sarcomere is the basic unit of muscle structure and function, and the sarcomere length changes with muscle fibre shrinkage or relaxation [6]. The myostatin (*MSTN*) gene encodes for a critical cytokine that negatively regulates skeletal muscle growth, which leads to hyperplasia and muscle hypertrophy in muscle cells when the *MSTN* gene is mutated or deleted [7]. In the animal embryonic phase, the regulation of *MSTN* mainly inhibits the increase in the number of muscle fibres but inhibits muscle hypertrophy after birth [8,9].

This study’s objective was to evaluate whether the muscle fibre development status of broiler breeders’ offspring was affected by different maternal dietary energy levels to assess the role of energy restriction and determine its possible benefits concerning the growth performance of their offspring.

## MATERIALS AND METHODS

This study was approved by the Animal Care and Use Committee of Northeast Agricultural University. Animals used in these experiments were cared for under the guidelines stated in the Guide for the Care and Use of Agricultural Animals in Agricultural Research and Teaching of Heilongjiang Province in China.

### Birds and experimental design

A single factor design was adopted. A total of 480 20-week-old Arbor Acres (AA) healthy female broiler breeders, with an average body weight of 2.33±0.01 kg, were randomly divided into 4 treatment groups with 6 replicates and 20 broiler breeders for each replicate. The formal study began when the laying rate reached 5%. The birds were fed a regular energy density diet in the 100% energy group, and the restriction diet metabolic energy levels were 80%, 70%, and 50% of the regular energy density diet in the experimental groups, while the other nutrient levels were the same (Table 1). All 4 groups had the same restricted feeding. Each bird was housed in an individual cage measuring 48 cm×34 cm×39 cm. At the beginning of the laying period, the henhouse’s photoperiod was increased by an average of 0.5 h per week with artificial light up to a maximum of 16 h/d and then was maintained at this level by the combination of natural and artificial light.

At a laying rate of more than 65%, 37 weeks, approximately 300 eggs per treatment were randomly collected over 6 days. Eggs were stored in a separate room at 10°C until incubation. Eggs were incubated at a constant eggshell temperature of 37.8°C during the entire incubation period. Besides, 120 0-day-old female broilers at each energy level were randomly selected and divided into 6 replicates with 20 broilers for each replicate. All of the offspring were given the same diet (Table 2). The experimental phase with the offspring lasted for 49 days.

### Samples collection and preparation

One egg was randomly selected from each replicate of each treatment for sampling at the 15th, 17th, 19th, and 21st embryonic days.

The breast muscles from the left side of the body provided material for histological examination and were fixed in formal saline as soon after death as possible [9,11]. Samples were dehydrated in alcohol and finally embedded in paraffin wax [12]. Transverse sections 10 mm thick were cut, stained with haematoxylin and eosin, and mounted in the usual way [13]. Subsequently, 8 photomicrographs for fibre diameter measurements were taken using the AMG EVOS microscope’s particular morphometric function. In each muscle sample, more than fifty diameters were measured, and the densities were calculated. Ultrathin sections were made using the method of Gratta et al [14]. The breast muscles were gently stretched, and ice-cold fixative with 2.5% glutaraldehyde, pH 7.2, was then added to the muscle, which was sliced into 2 to 3 mm thick strips parallel to the muscle fibres. The muscle strips were fixed at 4°C for 2 h and then further dissected in the fixative to blocks of 1 to 2 mm dimensions. These blocks were washed overnight at 4°C in 0.10 M phosphate buffer, pH 7.2. The sample was then postfixed in 2% osmium tetroxide for 1.5 h at 4°C, dehydrated through a graded series of ethanols and infiltrated with Epon 812. Semithin sections without counterstaining were inspected under a light microscope. Ultrathin sections from selected areas of each block, stained with uranyl acetate and lead citrate, were examined in a Hitachi H-7650 electron microscope operated at 60 kV.

Total RNA was isolated from the above samples according to standard TRIzol extraction procedures. The quality of total RNA was examined by ethidium bromide-stained denaturing agarose gel electrophoresis. The concentration of RNA was determined by a spectrophotometer. RNA samples were stored at −80°C before use. The total RNA of each sample was reverse transcribed using the PrimeScript RT reagent Kit (TaKaRa Biotechnology Co., Ltd. Dalian, China) according to the manufacturer’s instructions [11,15]. The polymerase chain reaction primers were designed according to the sequence of those genes in chickens in GenBank (Table 3) by Primer 5.0 and synthesized by the Beijing BGI Company, China. Each reaction was carried out in a total volume of 20 μL, consisting of 10.0 μL SYBR Premix Ex, 0.8 μL each primer, 2.0 μL cDNA and 6.0 μL ddH_2_O. Amplification reactions in triplicate for each sample were performed, and the results were normalized to the glyceraldehyde-3-phosphate dehydrogenase (*GAPDH*) gene expression level.

### Statistical analysis

Statistical analyses were performed using Statistical Analysis System (SAS) version 9.4. One-way analysis of variance (ANOVA) was used for statistical analysis. Data are expressed as means±standard error of the mean. Statistical comparisons of different treatments were performed using one-way ANOVA. The test results of all analyses were considered significant when p<0.05.

## RESULTS

**Figure 1:**
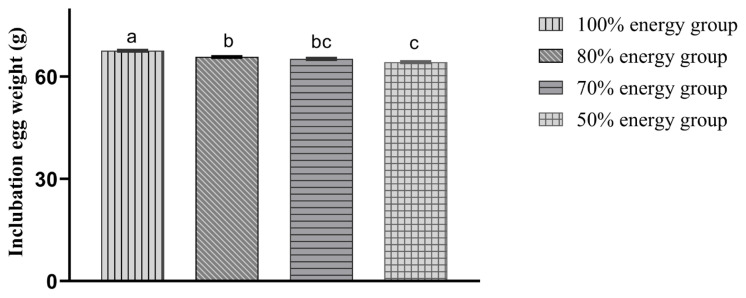
Comparison of incubation egg weight in different treatment groups. At a laying rate of more than 65%, 37 weeks, approximately 300 eggs per treatment were randomly collected over 6 days. Eggs were stored in a separate room at 10°C until incubation. And the incubation egg weight (g) in 100%, 80%, 70%, and 50% energy groups were 67.61±0.21^a^, 65.79±0.21^b^, 65.23±0.23^bc^, 64.25±0.22^c^, individually. The incubation egg weights in the 80%, 70%, and 50% energy groups were significantly lower (p<0.05) than those in the 100% energy group. Moreover, the hatching egg weight in the 50% energy group was significantly lower (p<0.05) than those in the 80% and 70% energy groups.

With decreasing energy levels, the incubation egg weight was also reduced accordingly. The incubation egg weights in the 80%, 70%, and 50% energy groups were significantly lower (p<0.05) than those in the 100% energy group. Moreover, the hatching egg weight in the 50% energy group was significantly lower (p<0.05) than those in the 80% and 70% energy groups. There was a decreasing trend (p>0.05) compared with the 80% and 70% energy groups (Figure 1).[Fig f2-ab-20-0712]

The diameters and density of the breast muscle fibres de creased gradually with decreasing dietary energy levels. Compared with the 100% energy group, the breast muscle fibre diameter at each embryonic day in the 50% energy group, at embryonic days 15, 17, and 21 in the 70% energy group, and at embryonic day 21 in the 80% energy group were significantly reduced (p<0.05), and there were no significant differences (p>0.05) among the other embryonic days. In the 80% energy group, the muscle fibre density of the breast increased significantly (p<0.05) at embryonic days 15 and 21, but there was no significant difference (p>0.05) at embryonic days 17 and 19. The breast muscle fibre density in the 70% and 50% energy groups was significantly increased (p<0.05) compared with that in the 100% energy group (Table 4).

Compared with the 100% energy group, the breast muscle fibre diameter of offspring in each group was significantly decreased (p<0.05) on the 1st day, and the 80% energy group was significantly higher (p<0.05) than the 50% energy group. There was no significant difference (p>0.05) among the four groups on the 28th day. Moreover, the 80% energy group’s breast muscle fibre diameter tended to increase (p>0.05). However, the 50% energy group showed an opposite tendency, and the 80% energy group was significantly higher (p<0.05) than the 50% energy group on the 49th day (Table 5).

The breast muscle fibre density of the offspring, compared with the 100% energy group, was significantly increased (p< 0.05) on the 1st day, and the 70% and 50% energy groups were significantly higher (p<0.05) than the 80% energy group. On the 28th day, the 70% and 50% energy groups showed a significant increase (p<0.05), but the 80% energy group showed no significant change (p>0.05). The breast muscle fibre density in the 70% energy group was significantly increased (p<0.05); the 80% energy group showed a decreasing trend (p>0.05), and the 50% energy group showed a tendency to increase (p>0.05), but neither reached a significant level. The breast muscle fibre density in the 70% energy group was significantly higher (p<0.05) than the 80% and 50% energy groups, and that of the 50% energy group was significantly higher (p<0.05) than the 80% energy group on the 49th day (Table 5).

The breast muscle sarcomere length of the embryos, com pared with the 100% energy group, showed an increasing trend (p>0.05) at the 15th and 17th embryonic days in the 80% energy group, and the 70% and 50% energy groups showed an opposite tendency (p>0.05). However, none of these reached a significant level, with the 80% energy group significantly higher (p<0.05) than the 70% and 50% energy groups. At the 19th embryonic day, the lengths in the 50% and 70% energy groups were significantly decreased (p<0.05), and that of the 80% energy group showed no significant (p> 0.05) change. On the 21st day, the length in the 80% energy group was significantly increased (p<0.05), those of the 70% and 50% energy groups were significantly reduced (p<0.05), and that of the 80% energy group was significantly higher (p<0.05) than the 70% and 50% energy groups (Table 6).

Compared with the 100% energy group, the expression of the *MSTN* gene in the offspring was significantly decreased (p<0.05), but there was no significant difference (p>0.05) among the maternal dietary energy restriction groups on the 49th day.

## DISCUSSION

Maternal nutrition is an essential factor for the growth and development of offspring; the chicken’s weight is usually 62% to 76% of the hatching egg weight initially, and there is a strong positive correlation between incubation egg weight and chicken weight [1,2,16–19]. In this study, it was found that the energy-limited diet of broiler chickens significantly reduced the weight of the incubated eggs. This then reduced the offspring’s birth weight, which was more consistent with Pinchasov’s research results [1,13].

In this study, the diameter of breast muscle fibres increased with embryonic age, which indicated that muscle fibre differentiation was accompanied by muscle fibre hypertrophy. The breast muscle fibre density first increased and then decreased, which indicated the continuous differentiation of muscle fibres. This phenomenon continued into the 17th to 19th embryonic days, which was consistent with Picard B’s reports [20]. After that, the breast muscle fibre diameter continued to increase. The muscle fibre density began to decrease, indicating that the differentiation of the breast muscle fibres was completed or reduced at this time, which decreased the muscle fibre density per unit area [21].

**Figure 3:**
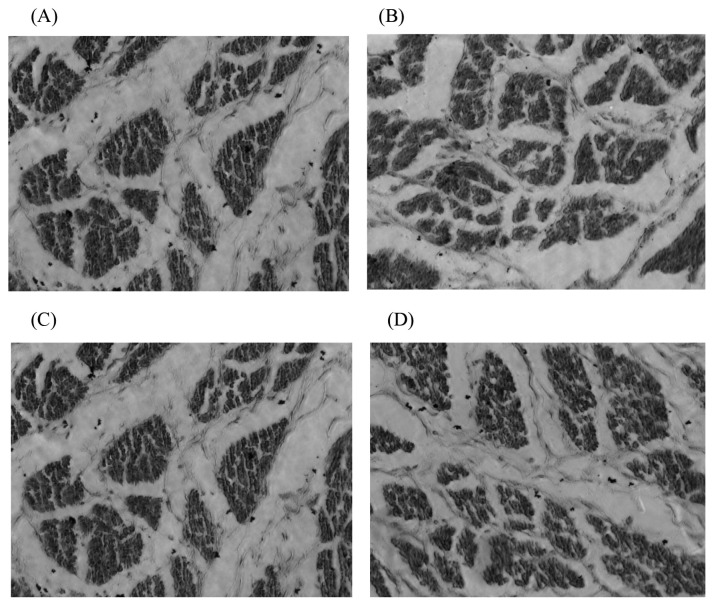
The paraffin sections of breast muscle fiber in the 15th day embryo in 400× AMG EVOS microscope. (A) 100% energy group; (B) 80% energy group; (C) 70% energy group; (D) 50% energy group. Breast muscles were dehydrated in alcohol and finally embedded in paraffin wax. Transverse sections 10 mm thick were cut, stained with haematoxylin and eosin, and mounted in the usual way. Subsequently, 8 photomicrographs for fibre diameter measurements were taken using the AMG EVOS microscope’s particular morphometric function. In each muscle sample, more than fifty diameters were measured, and the densities were calculated. Compared with the 100% energy group, in general, the diameter and density of the embryonic breast muscle fibres in each energy-restricted group showed a decreasing trend.

Compared with the 100% energy group, in general, the diameter and density of the embryonic breast muscle fibres in each energy-restricted group showed a decreasing trend (Figure 3). The offspring embryonic muscle fibre diameter was significantly reduced by maternal dietary energy restriction in the 70% and 50% energy groups. This result suggested that muscle fibre growth was restricted, which may be associated with the offspring’s lower birth weight. The smaller muscle fibre diameter might result in a larger muscle fibre density. The offspring embryo muscle fibre changes were smaller in the 80% energy group, which indicated a lower degree of the effect of maternal dietary energy restriction on offspring embryonic muscle fibre development, and excessively high maternal dietary energy restriction had a detrimental effect on muscle fibre development.

**Figure 4:**
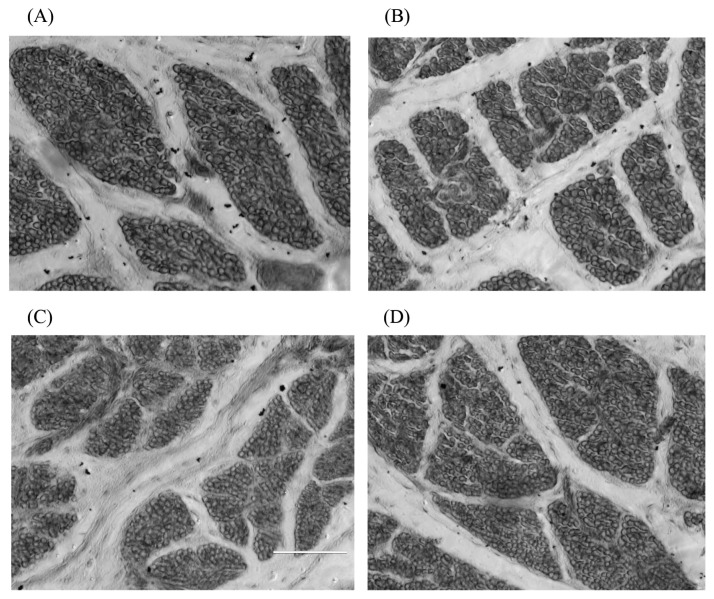
The paraffin sections of breast muscle fiber in the 1st day offspring in 400× AMG EVOS microscope. (A) 100% energy group; (B) 80% energy group; (C) 70% energy group; (D) 50% energy group. Breast muscles were dehydrated in alcohol and finally embedded in paraffin wax. Transverse sections 10 mm thick were cut, stained with haematoxylin and eosin, and mounted in the usual way. Subsequently, 8 photomicrographs for fibre diameter measurements were taken using the AMG EVOS microscope’s particular morphometric function. In each muscle sample, more than fifty diameters were measured, and the densities were calculated. This study showed that maternal dietary energy restriction could significantly reduce the breast muscle fibre diameter of 1st day offspring and significantly increase their muscle fibre density.

**Figure 5:**
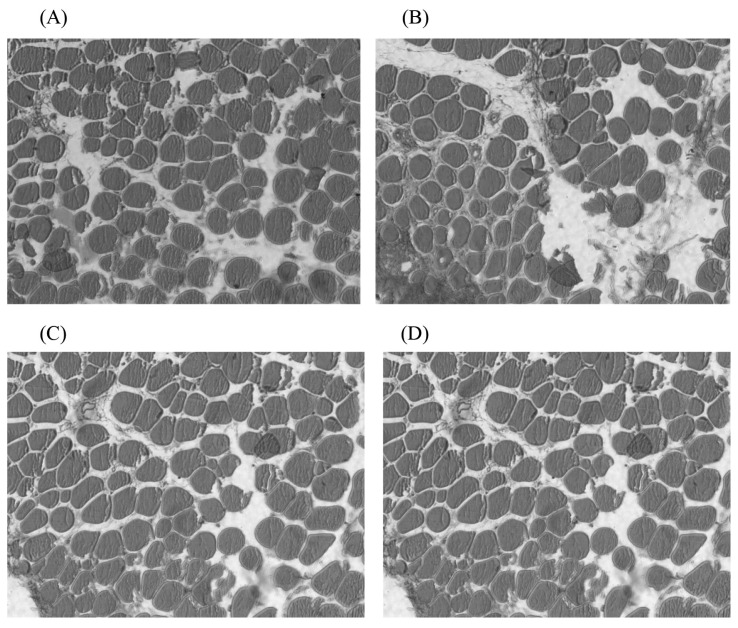
The paraffin sections of breast muscle fiber in the 49th day offspring in 400× AMG EVOS microscope. (A) 100% energy group; (B) 80% energy group; (C) 70% energy group; (D) 50% energy group. Breast muscles were dehydrated in alcohol and finally embedded in paraffin wax. Transverse sections 10 mm thick were cut, stained with haematoxylin and eosin, and mounted in the usual way. Subsequently, 8 photomicrographs for fibre diameter measurements were taken using the AMG EVOS microscope’s particular morphometric function. In each muscle sample, more than fifty diameters were measured, and the densities were calculated. On the 49th days, the diameter and density of breast muscle fibres in the offspring in the 80% energy group were not significantly different from those in the 100% energy group, indicating that there was a compensatory effect on muscle fibre growth, the breast muscle fibre density in the 70% and 50% energy groups was significantly increased or showed an increasing trend.

Xu et al [22] reported that maternal broiler breeders fed a low-energy diet had significantly reduced offspring muscle fibre diameters and significantly increased muscle fibre densities. Yan et al [23] found that low-protein diets could reduce muscle fibre diameter and increase the muscle fibre density in broiler breeders. This study showed that maternal dietary energy restriction could significantly reduce the breast muscle fibre diameter of 1st-day offspring and significantly increase their muscle fibre density (Figure 4), which confirmed the presence of maternal effects. On the 28th and 49th days, the diameter and density of breast muscle fibres in the offspring in the 80% energy group were not significantly different from those in the 100% energy group (Figure 5), indicating that there was a compensatory effect on muscle fibre growth, while in 70% and 50% energy level groups, the offspring’s breast muscle fibre density was significantly reduced on the 28th day. On the 49th day, the breast muscle fibre density in the 70% and 50% energy groups was significantly increased or showed an increasing trend, which may be related to the smaller muscle fibre diameter. Different energy groups showed different trends, which may be caused by energy changes in the broiler breeder’s diet, which affected the deposition of nutrients in the eggs and eventually resulted in differences in the muscle fibre development process. The results showed that the maternal diet’s 80% energy level had no significant effect on the muscle fibre traits of the offspring. In contrast, the higher energy restriction of the maternal broiler breeder hens significantly reduced the muscle fibre diameter of the offspring, which might harm the development of the offspring’s muscle fibres.

At present, little is known about maternal nutrition’s effect on the sarcomere length of broiler chicks. Shan found that limited broiler feed intake had no significant effect on the sarcomere length of the breast muscle and leg muscle of offspring broilers on the 56th day [24]. This experiment found that breast muscle’s sarcomere length increased with embryonic age until hatching in each group. After hatching, the sarcomere length continues to increase, reaching its maximum length on the 28th day and decreasing on the 49th day, which was similar to the results of Song’s study on piglets [25]. The increase in sarcomere length may be attributed to the rapid increase in the synthesis of myosin and actin, which accelerates the formation of rough myofilaments mainly composed of myosin and of fine myofilaments mainly composed of actin, thus increasing the number of rough and fine myofilaments and making the myofibrils longer and thicker. The thickening of the myofibrils led to the thickening of muscle fibres, which was an essential factor in increasing muscle fibre diameter, as the sarcomere length declined on the 49th day, which might be related to slaughter stress. Compared with these 4 groups, the effect of maternal dietary energy restriction on the embryonic sarcomere of the offspring was small, especially in the 80% energy group, in which the sarcomere length of the embryos was significantly increased on the 21st day. There was no significant change in the embryo breast muscle’s sarcomere length on the other days, which indicated that a lower level of energy restriction was without adverse effects on the muscle fibre development of offspring.

This study of sarcomere length of the offspring found that a maternal energy restriction diet significantly increased the sarcomere length of the breast muscle in the offspring in the 80% energy group and significantly reduced those in the 70% and 50% energy group on the 1st day but had no significant effect on the 28th and 49th day. The results of this experiment showed significant tissue differences, which may be caused by dietary energy restriction of the broiler breeders leading to changes in maternal metabolism, which further affected the deposition of nutrients in the eggs and finally led to differences in the development process and morphology of the muscle fibres in the offspring.

Xu et al [26] found that low-energy maternal diets signifi cantly reduced *MSTN* gene expression on the 1st day in offspring and had no significant effect on the 28th and 56th days. In this study, the relative expression of the *MSTN* gene in the offspring was significantly lower than that in the 100% energy group on the 49th day, suggesting that maternal dietary energy restriction was beneficial to muscle fibre hypertrophy in the late growth stage. The 80% energy group was particularly prominent among them, which may be associated with a greater body weight at 49 days in the 80% energy group.

Myogenesis is regulated through the proliferation and dif ferentiation of myoblasts expressing *MSTN*, which functions as a negative regulator by generating Smad signals [27].

Myostatin is a member of the transforming growth factor-β (*TGF-β*) superfamily, which plays a vital role in the proper regulation of skeletal muscle growth. During embryogenesis, *MSTN* is expressed in the myotome and developing skeletal muscles [28] and acts to regulate the final number of muscle fibres. Mutations in the *MSTN* gene in mice [29] and the double-muscled phenotype in goats [30] display a drastic increase in skeletal muscle mass, indicating a function for myostatin as a negative regulator of muscle growth.

It is inferred that the differential expression of MSTN might or at least partially cause muscle development differences in the offspring from the different groups.

## CONCLUSION

These results indicate that the muscle fibre diameter, density, and sarcomere length of offspring were significantly affected by maternal dietary energy restriction in the 70% and 50% energy groups, while the 80% energy group was less affected and improved at 49 days. Maternal energy restriction significantly reduced the relative expression of MSTN mRNA in offspring on the 49th day. There were significant maternal effects and compensatory growth effects on the breast muscle fibre growth of offspring. In conclusion, the effects of a maternal dietary energy level of 80% in this study were optimal for breast muscle fibre development in offspring, which indicated that the metabolic energy level of AA broilers of 9.36 MJ/kg for the mid-term diet for laying eggs has a practical significance.

## Figures and Tables

**Figure 2 f2-ab-20-0712:**
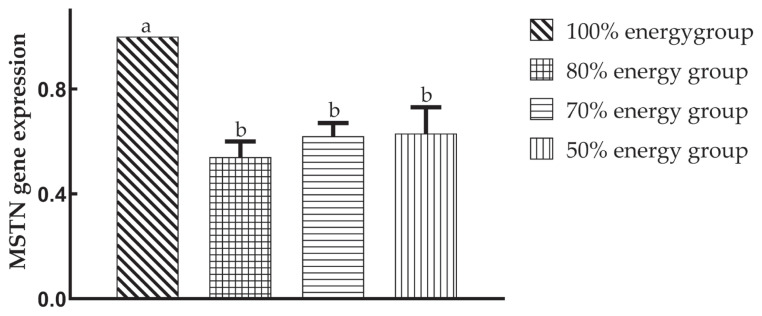
Effects of maternal dietary energy restriction on the relative expression of the myostatin (*MSTN*) gene of offspring breast muscles on the 49th day. Total RNA was isolated from the left side breast muscles according to standard TRIzol extraction procedures. The quality of total RNA was examined by ethidium bromide-stained denaturing agarose gel electrophoresis. The concentration of RNA was determined by a spectrophotometer. RNA samples were stored at −80°C before use. The polymerase chain reaction primers were designed according to the sequence of those genes in chickens in GenBank by Primer 5.0 and synthesized by the Beijing BGI Company, China. Each reaction was carried out in a total volume of 20 μL, consisting of 10.0 μL SYBR Premix Ex, 0.8 μL each primer, 2.0 μL cDNA and 6.0 μL ddH_2_O. Amplification reactions in triplicate for each sample were performed, and the results were normalized to the *GAPDH* gene expression level. Compared with the 100% energy group (1.00±0.00^a^), the expression of the *MSTN* gene in the offspring was significantly decreased (p<0.05), but there was no significant difference (p>0.05) among the maternal dietary energy restriction groups, 0.54±0.06^b^, 0.62±0.05^b^, 0.63±0.10^b^, individually, on the 49th day.

**Table 1:** Composition (kg/100 kg) of diets^1)^ for broiler breeders during the laying period

Items	100% energy group	80% energy group	70% energy group	50% energy group
Ingredients				
Corn	62.04	48.00	37.98	18.00
Soybean meal	26.10	29.20	31.08	34.98
Soybean oil	2.00			
NaCl	0.17	0.17	0.17	0.17
CaHPO_4_	1.50	1.60	1.65	1.80
Limestone	7.70	7.60	7.60	7.50
DL-methionine	0.06	0.08	0.09	0.12
Choline chloride	0.10	0.10	0.10	0.10
Premix^2)^	0.33	0.33	0.33	0.33
Rice hull		12.92	21.00	37.00
Total (kg)	100.00	100.00	100.00	100.00
Nutritive values, on air-dry basis				
Metabolic energy^3)^ (MJ/kg)	11.70	9.36	8.19	5.85
Crude protein^4)^ (%)	17.01	17.01	17.02	17.00
Lysine^4)^ (%)	0.80	0.81	0.81	0.82
Methionine (%)	0.34	0.34	0.34	0.34
Calcium^4)^ (%)	3.30	3.30	3.30	3.30
Phosphorus^4)^ (%)	0.45	0.45	0.45	0.45

1) Based on the National Research Council (NRC) [10] nutrient requirements for broiler breeders during the laying period.

2) The premix provided the following per kg of diets: Vit A 12,000 IU, Vit D 2,400 IU, Vit E 30 IU, Vit K 1.5 mg, Vit B12 0.012 mg, Vit B1 2.0 mg, riboflavin 9 mg, pantothenic acid 12 mg, nicotinic acid 35 mg, pyridoxine 4.5 mg, biotin 0.20 mg, folic acid 1.2 mg, Fe 80 mg, Cu 8 mg, Mn 100 mg, Zn 80 mg, I 1.0 mg, Se 0.30 mg.

3) Calculated values (NRC [10]).

4) Analysed content.

**Table 2:** Composition (kg/100 kg) of diets^1)^ for broiler breeders’ offspring

Items	1 to 3 wk	4 to 6 wk	7 wk
Ingredients			
Corn	61.69	66.30	69.90
Soybean meal	31.00	25.47	21.90
Fish meal	2.00	3.00	2.00
Soybean oil	2.00	2.50	3.00
Salt	0.17	0.12	0.12
CaHPO_4_	1.20	0.90	0.75
Limestone	1.30	1.20	1.15
DL-methionine	0.21	0.08	0.75
Choline chloride	0.10	0.10	0.10
Premix	0.33^2)^	0.33^3)^	0.33^4)^
Total (kg)	100.00	100.00	100.00
Nutritive values, on air-dry basis			
Metabolic energy^5)^ (MJ/kg)	12.54	12.96	13.17
Crude protein^6)^ (%)	21.51	19.98	18.00
Lysine^6)^ (%)	1.18	1.08	0.93
Methionine+cysteine^6)^ (%)	0.92	0.76	0.66
Calcium^6)^ (%)	1.02	0.91	0.80
Phosphorus^6)^ (%)	0.46	0.42	0.35

1) Based on the NRC [10] nutrient requirements for broiler breeders during the different growing period.

2) The premix provided the following per kg of diets: Vit A 8,000 IU, Vit D 1,000 IU, Vit E 20 IU, Vit K 0.5 mg, thiamine 2.0 mg, riboflavin 8 mg, pantothenic acid 10 mg, nicotinic acid 35 mg, pyridoxine 3.5 mg, biotin 0.18 mg, folic acid 0.55 mg, Vit B_12_ 0.010 mg, Fe 100 mg, Cu 8 mg, Mn 120 mg, Zn 100 mg, I 0.7 mg, Se 0.3 mg.

3) The premix provided the following per kg of diets: Vit A 6,000 IU, Vit D 750 IU, Vit E 10 IU, Vit K 0.5 mg, thiamine 2.0 mg, riboflavin 5 mg, pantothenic acid 10 mg, nicotinic acid 30 mg, pyridoxine 3.0 mg, biotin 0.15 mg, folic acid 0.55 mg, Vit B_12_ 0.010 mg, Fe 80 mg, Cu 8 mg, Mn 100 mg, Zn 80 mg, I 0.7 mg, Se 0.3 mg.

4) The premix provided the following per kg of diets: Vit A 2,700 IU; Vit D 400 IU, Vit E 10 IU, Vit K 0.5 mg, thiamine 2.0 mg, riboflavin 5 mg, pantothenic acid 10 mg, nicotinic acid 30 mg, pyridoxine 3.0 mg, biotin 0.10 mg, folic acid 0.50 mg, Vit B_12_ 0.007 mg, Fe 80 mg, Cu 8 mg, Mn 80 mg, Zn 80 mg, I 0.7 mg, Se 0.3 mg.

5) Calculated values (NRC [10]).

6) Analysed content.

**Table 3:** Primer sequences and GenBank accession number for the target genes

Target gene	Primer sequence (5′-3′)	Product length/bp	Acc. No.^1)^
*GAPDH*	PF: 5′-GCCATCACAGCCACACAGA-3′ PR: 5′-TTTCCCCACAGCCTTAGCA-3′	120	NM_204305
*MSTN*	PF: 5′-TTTTGGATGGGACTGGATTATAGCACCT-3′ PR: 5′-GCCTCTGGGATTTGCTTGGTGTACC-3′	127	NM_001001461.1

PF, forward primer; FR, reverse primer; *GAPDH*, glyceraldehyde-3-phosphate dehydrogenase; *MSTN*, myostatin.

1) Acc. No: GenBank accession number. Primers were designed according to the sequence of the target genes by there Acc. No. in GenBank.

**Table 4:** Effects of maternal dietary energy restriction on diameter and density of breast muscle fiber in the embryo

Items	Embryo age (d)	100% energy group	80% energy group	70% energy group	50% energy group	p-value
Diameter (μm)	15	7.16±0.21^a^	6.75±0.47^a^	5.84±0.19^b^	5.24±0.21^b^	0.0415
	17	7.57±0.27^a^	7.23±0.13^ab^	6.50±0.25^c^	5.90±0.15^c^	0.0331
	19	7.28±0.18	7.14±0.49	6.91±0.32	7.28±0.13	0.0657
	21	7.39±0.36^b^	9.73±1.18^a^	7.37±.31^b^	7.39±0.39^b^	0.0401
Density (N/mm^2^)	15	9,864±202.31^d^	11,745±287.86^c^	1,445±455.12^b^	17,541±452.92^a^	0.0305
	17	11,287±406.51^c^	12,258±313.11^bc^	13,423±271.24^b^	19,615±474.69^a^	0.0415
	19	11,581±194.64^c^	12,307±479.31^bc^	13,874±254.57^b^	19,046±894.96^a^	0.0207
	21	10,471±840.02^c^	11,843±324.23^b^	11,220±190.93^bc^	15,425±229.19^a^	0.0471

a–d Values with different letter superscripts in the same row mean significant difference (p<0.05), while with the same or no letter superscripts mean no significant difference (p>0.05).

**Table 5:** Effects of maternal dietary energy restriction on diameter and density of breast muscle fiber in the offspring

Items	Embryo age (d)	100% energy group	80% energy group	70% energy group	50% energy group	p-value
Diameter (μm)	1	8.87±0.15^a^	7.94±0.09^b^	7.49±0.25^bc^	6.60±0.59^c^	0.0351
	28	26.87±1.54^b^	26.14±0.49^b^	29.61±0.78^a^	31.34±0.86^a^	0.0237
	49	43.10±2.67^ab^	44.03±0.77^a^	40.39±1.38^ab^	39.24±1.37^b^	0.0339
Density (N/mm^2^)	1	946±194.98^b^	9,033±295.92^b^	11,637±302.20^a^	12,049±278.46^a^	0.0211
	28	988±12.42^b^	1,009±14.29^b^	774±24.91^a^	809±17.78^a^	0.0424
	49	384±4.83^bc^	362±6.85^c^	466±6.96^a^	398±9.61^b^	0.0327

a–c Values with different letter superscripts in the same row mean significant difference (p<0.05), while with the same or no letter superscripts mean no significant difference (p>0.05).

**Table 6:** Effects of maternal dietary energy restriction on breast sarcomere length of embryo

Embryo age (d)	100% energy group	80% energy group	70% energy group	50% energy group	p-value
11	1,257±36.15	1,280±22.03	1,212±36.37	1,220±21.62	0.07152
13	1,287±35.50	1,312±21.08	1,233±24.75	1,252±53.20	0.0655
15	1,343±25.09^ab^	1,377±31.49^a^	1,261±42.16^b^	1,271±31.16^b^	0.0415
17	1,382±37.46^ab^	1,410±16.86^a^	1,277±28.74^b^	1,280±25.69^b^	0.0377
19	1,419±18.09^a^	1,452±30.22^a^	1,292±37.82^b^	1,277±28.58^b^	0.0422
21	1,448±27.28^b^	1,504±36.53^a^	1,303±25.70^c^	1,320±10.57^c^	0.0392

a–c Values with different letter superscripts in the same row mean significant difference (p<0.05), while with the same or no letter superscripts mean no significant difference (p>0.05).

## References

[1] Senbeta EK (2017). Effect of egg size on hatchability and subsequent growth performance of fayoumi chicken. J Agric Sci.

[2] Goliomytis M, Skoupa EP, Konga A, Symeon GK, Charismiadou MA, Deligeorgis SG (2016). Influence of gestational maternal feed restriction on growth performance and meat quality of rabbit offsprings. Animal.

[3] Li F, Xu LM, Shan AS, Hu JW, Zhang YY, Li YH (2011). Effect of daily feed intake in laying period on laying performance, egg quality and egg composition of genetically fat and lean lines of chickens. Br Poult Sci.

[4] Koomkrong N, Theerawatanasirikul S, Boonkaewwan C, Jaturasitha S, Kayan A (2015). Breed-related number and size of muscle fibres and their response to carcass quality in chickens. Ital J Anim Sci.

[5] Verdiglione R, Cassandro M (2013). Characterization of muscle fiber type in the pectoralis major muscle of slow-growing local and commercial chicken strains. Poult Sci.

[6] Attia YA, Abd-ElHamid AEE, Mustafa M, Al-Harthi MA, Muhammad M (2017). Response of slow growing chickens to feed restriction and effects on growth performance, blood constituents and immune markers. Rev Mex Cienc Pecu.

[7] Aiello D, Patel K, Lasagna E (2018). The myostatin gene: an overview of mechanisms of action and its relevance to livestock animals. Anim Genet.

[8] Trukhachev V, Belyaev V, Kvochko A (2015). Myostatin gene (MSTN) polymorphism with a negative effect on meat productivity in Dzhalginsky Merino sheep breed. J BioSci Biotechnol.

[9] Lee SJ, Lehar A, Liu Y (2020). Functional redundancy of type I and type II receptors in the regulation of skeletal muscle growth by myostatin and activin A. Proc Natl Acad Sci USA.

[10] NRC (1994). Nutrient requirement of poultry.

[11] Attia YA, El-Tahawy WS, Abd El-Hamid AHE (2014). Effect of feed form, pellet diameter and enzymes supplementation on carcass characteristics, meat quality, blood plasma constituents and stress indicators of broilers. Arch Anim Breed.

[12] Wester K, Wahlund E, Sundström C (2000). Paraffin section storage and immunohistochemistry. Effects of time, temperature, fixation, and retrieval protocol with emphasis on p53 protein and MIB1 antigen. Appl Immunohistochem Mol Morphol.

[13] Ban Q, Liang Y, Zhao Z, Liu X, Li Q (2013). Differential expression levels of genes related to myogenesis during embryogenesis of quail and chicken. Pak Vet J.

[14] Gratta F, Birolo M, Sacchetto R (2019). Effect of feed restriction timing on live performance, breast myopathy occurrence, and muscle fiber degeneration in 2 broiler chicken genetic lines. Poult Sci.

[15] Livak KJ, Schmittgen TD (2001). Analysis of relative gene expression data using real-time quantitative PCR and the 2(-Delta delta C(T)) method. Methods.

[16] Al-Nedawi AM, Aljanabi TK, Altaie SM, Al-Samarai FR (2019). Effect of sex and day-old weight on subsequent body weight and body mass index in commercial broilers. Adv Anim Vete Sci.

[17] Li F, Mou SY, Liu Y (2018). Maternal dietary energy levels affected the lipid deposition of offspring embryos at the end of the laying period of broiler breeder hens. Ital J Anim Sci.

[18] Li F, Shan MX, Gao X (2019). Effects of nutrition restriction of fat-and lean-line broiler breeder hens during the laying period on offspring performance, blood biochemical parameters, and hormone levels. Domest Anim Endocrinol.

[19] Li F, Yang Y, Yang X (2019). Dietary intake of broiler breeder hens during the laying period affects amino acid profiles in eggs. R Bras Zootec.

[20] Picard B, Lefaucheur L, Berri C, Duclos MJ (2002). Muscle fibre ontogenesis in farm animal species. Reprod Nutr Dev.

[21] Kellermeier JD, Tittor AW, Brooks JC (2009). Effects of zilpaterol hydrochloride with or without an estrogen-trenbolone acetate terminal implant on carcass traits, retail cutout, tenderness, and muscle fiber diameter in finishing steers. J Anim Sci.

[22] Xu LM, Chen ZH, Li F, Cheng BJ, Shan AS (2010). Effects of maternal dietary energy restriction on growth performance, carcass and meat quality of broilers. Chinese J Anim Nutr.

[23] Yan J, Shan A, Shi B, Wang A, Hu J (2009). Influence of different protein level of maternal diets on muscle fiber and expression of myostatin gene of offspring in broiler. Chinese Acta Vet et Zootec Sinica.

[24] Anshan Shan, Feng Li (2012). Effect of maternal nutrition on offspring growth, development and meat quality in chicken. Chinese J Northeast Agric Univ.

[25] Song CY, Gao B, Jing RB, Tao Y, Mao JD (2003). Study on pig growth hormone gene polymorphisms in western meat-type breeds and Chinese local breeds. J Zhejiang Univ Sci.

[26] Xu L, Lu L, Zhang H (2012). Effect of energy restriction on growth performance, serum biochemicalindices and sarcomere length of offspring in broiler breeders during themedium laying period. J Northeast Agric Univ.

[27] Kurokawa M, Sato F, Aramaki S, Soh T, Yamauchi N, Hattori M (2009). Monitor of the myostatin autocrine action during differentiation of embryonic chicken myoblasts into myotubes: effect of IGF-I. Mol Cell Biochem.

[28] Bi Y, Feng B, Wang Z (2020). Myostatin (MSTN) gene indel variation and its associations with body traits in shaanbei white cashmere goat. Animals.

[29] Sugg KB, Korn MA, Sarver DC, Markworth JF, Mendias CL (2017). Inhibition of platelet-derived growth factor signaling prevents muscle fiber growth during skeletal muscle hypertrophy. FEBS Lett.

[30] He Z, Zhang T, Lei J (2018). Use of CRISPR/Cas9 technology efficiently targetted goat myostatin through zygotes microinjection resulting in double-muscled phenotype in goats. Biosci Rep.

